# Supplementary material for: An open-like conformation of the sigma-1 receptor reveals its ligand entry pathway
Source: Nat Commun. 2022 Mar 10;13:1267. doi: 10.1038/s41467-022-28946-w (PMC8913746; doi:10.1038/s41467-022-28946-w)

Supplementary information for

An open-like conformation of the sigma-1 receptor reveals its ligand entry pathway

Fuhui Meng[1‡], Yang Xiao[1‡], Yujia Ji[1], Ziyi Sun[1*], Xiaoming Zhou[1*]

[1]Department of Integrated Traditional Chinese and Western Medicine, Rare Diseases Center, State Key Laboratory of Biotherapy, West China Hospital, Sichuan University, Chengdu, Sichuan 610041, China

[‡]These authors contributed equally to this work.

*To whom correspondence should be addressed:

Ziyi Sun, PhD, 17 Renmin Road South 3rd Section, Sichuan University, Chengdu, Sichuan 610041, China; Phone: +86 (28) 8540 1152; Email: ziyi.sun@scu.edu.cn

Xiaoming Zhou, PhD, 17 Renmin Road South 3rd Section, Sichuan University, Chengdu, Sichuan 610041, China; Phone: +86 (28) 8540 1152; Email: x.zhou@scu.edu.cn

Supplementary Figures and Legends

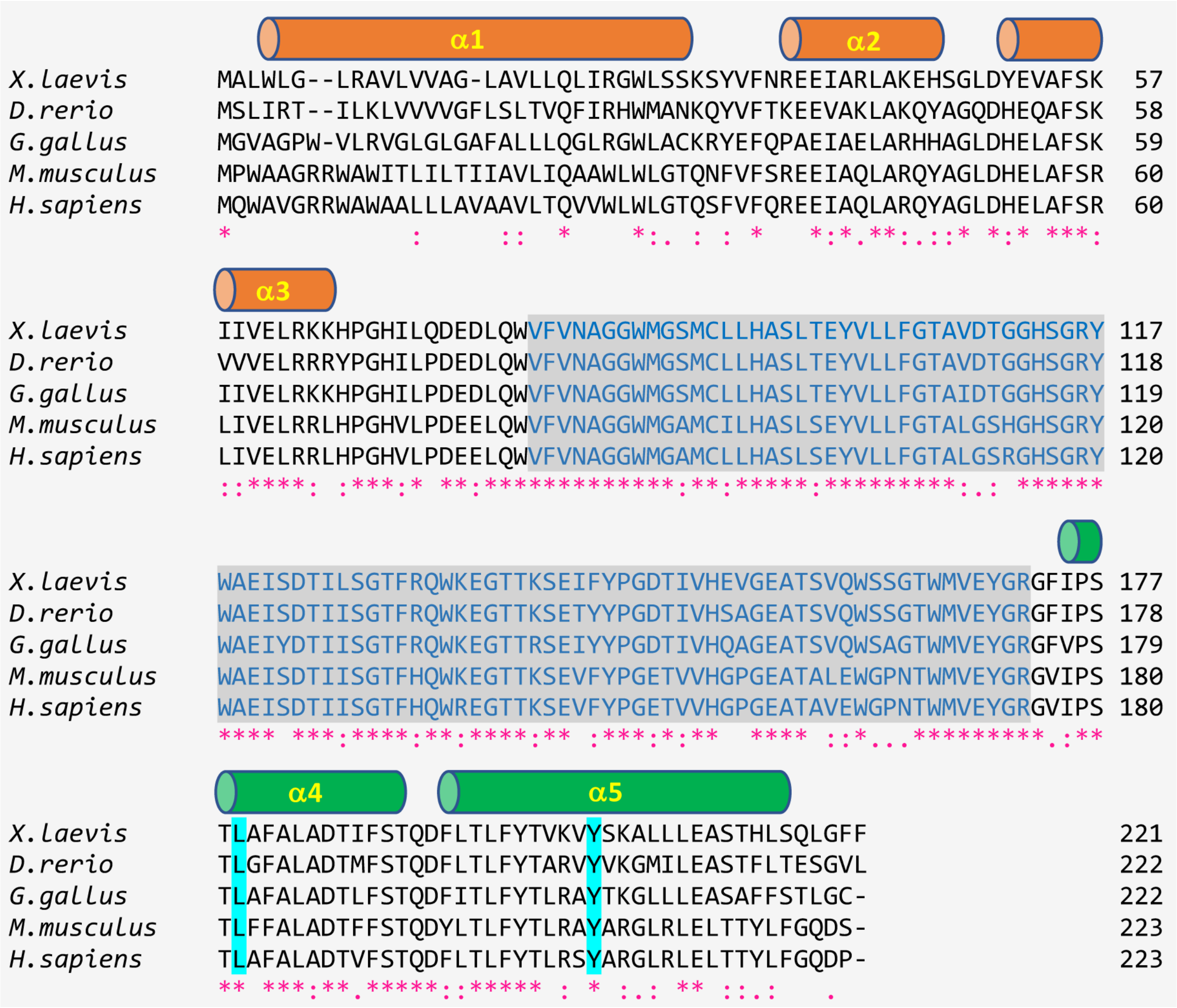

Supplementary Figure 1. Sequence alignment of σ1R homologs by ClustalW[1,2]. Alpha helices are indicated by cylinders and labeled from α1 to α5. The cupin-fold β barrel is in blue letters and highlighted in grey. Leu179 and Tyr203 of xlσ1R and their equivalent residues in other species are highlighted in cyan. Asterisks (*) indicate identical residues. Colons (:) indicate strong similarities. Periods (.) indicate weak similarities. *X. laevis, Xenopus laevis*; *D. rerio, Danio rerio*; *G. gallus, Gallus gallus*; *M. musculus, Mus musculus*; *H. sapiens, Homo sapiens*.

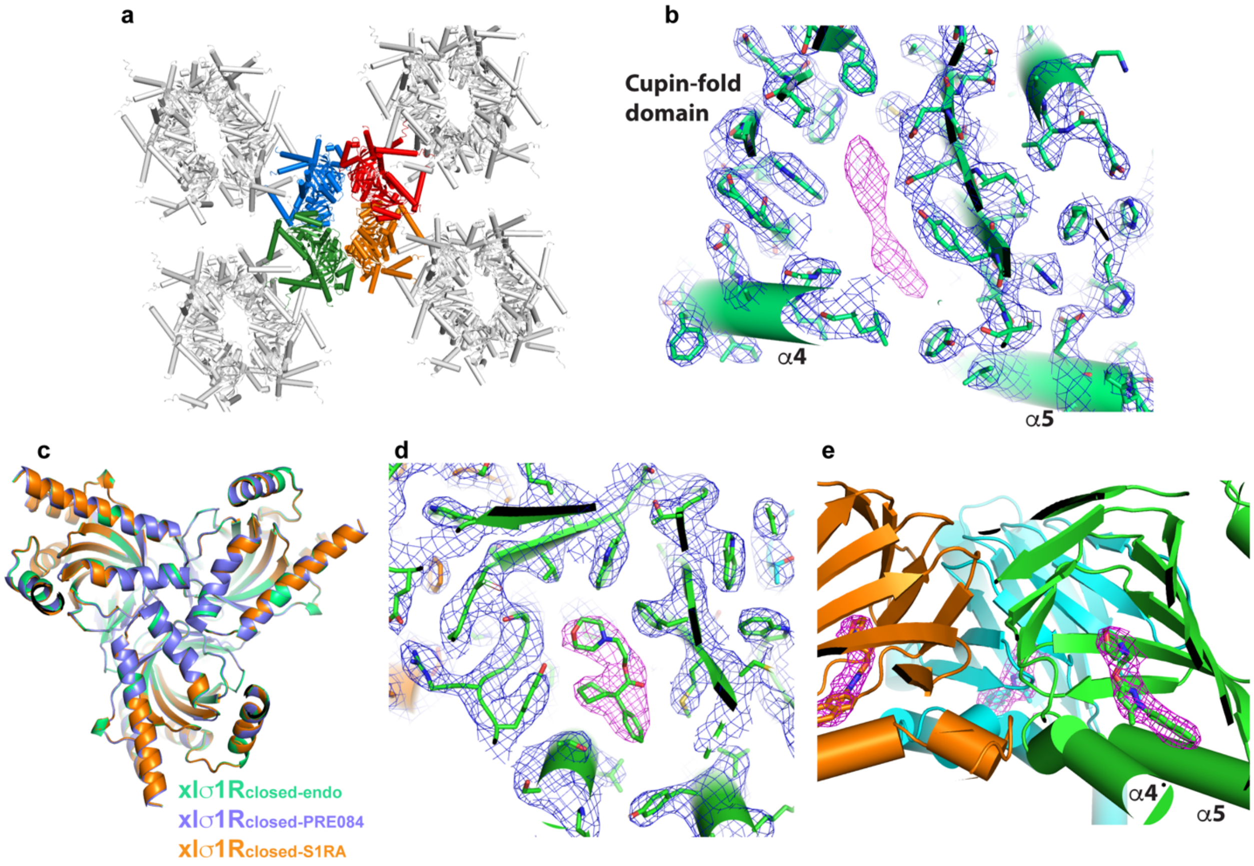

Supplementary Figure 2. The structures of xlσ1R in the closed conformation. (a) Crystal packing of $xl\sigma_1R_{closed-endo}$. The four homotrimers in one asymmetric unit are displayed in four colors, and four adjacent asymmetric units are displayed in grey. (b) The close-up view of the electron density map of one protomer of $xl\sigma_1R_{closed-endo}$, which is displayed in both sticks and the cartoon mode, viewed parallel to the membrane. The blue mesh shows the simulated annealing $2F_o$-F_c map contoured at 1.2 σ level, and the purple mesh shows the simulated annealing F_o-F_c map contoured at 3.0 σ level corresponding to an unidentifiable molecule. (c) Superposition of the $xl\sigma1R_{closed-endo}$ trimer (in green), the $xl\sigma1R_{closed-PRE084}$ trimer (in light blue), and the $xl\sigma1R_{closed-S1RA}$ trimer (in orange), viewed perpendicular to the membrane from the membrane side. (d) The close-up view of the electron density map of one protomer of $xl\sigma_1R_{closed-PRE084}$, which is displayed in both sticks and the cartoon mode, viewed parallel to the membrane. The blue mesh shows the simulated annealing $2F_o$-F_c map contoured at 1.2 σ level, and the purple mesh shows the

simulated annealing F_o-F_c omit map contoured at 3.0 σ level corresponding to PRE084 (in sticks). (e) The close-up view of the xl$\sigma_1 R_{closed\text{-}S1RA}$ trimer viewed parallel to the membrane. The ligand S1RA is displayed in sticks, and the purple mesh shows the simulated annealing F_o-F_c omit map contoured at 3.0 σ level corresponding to S1RA.

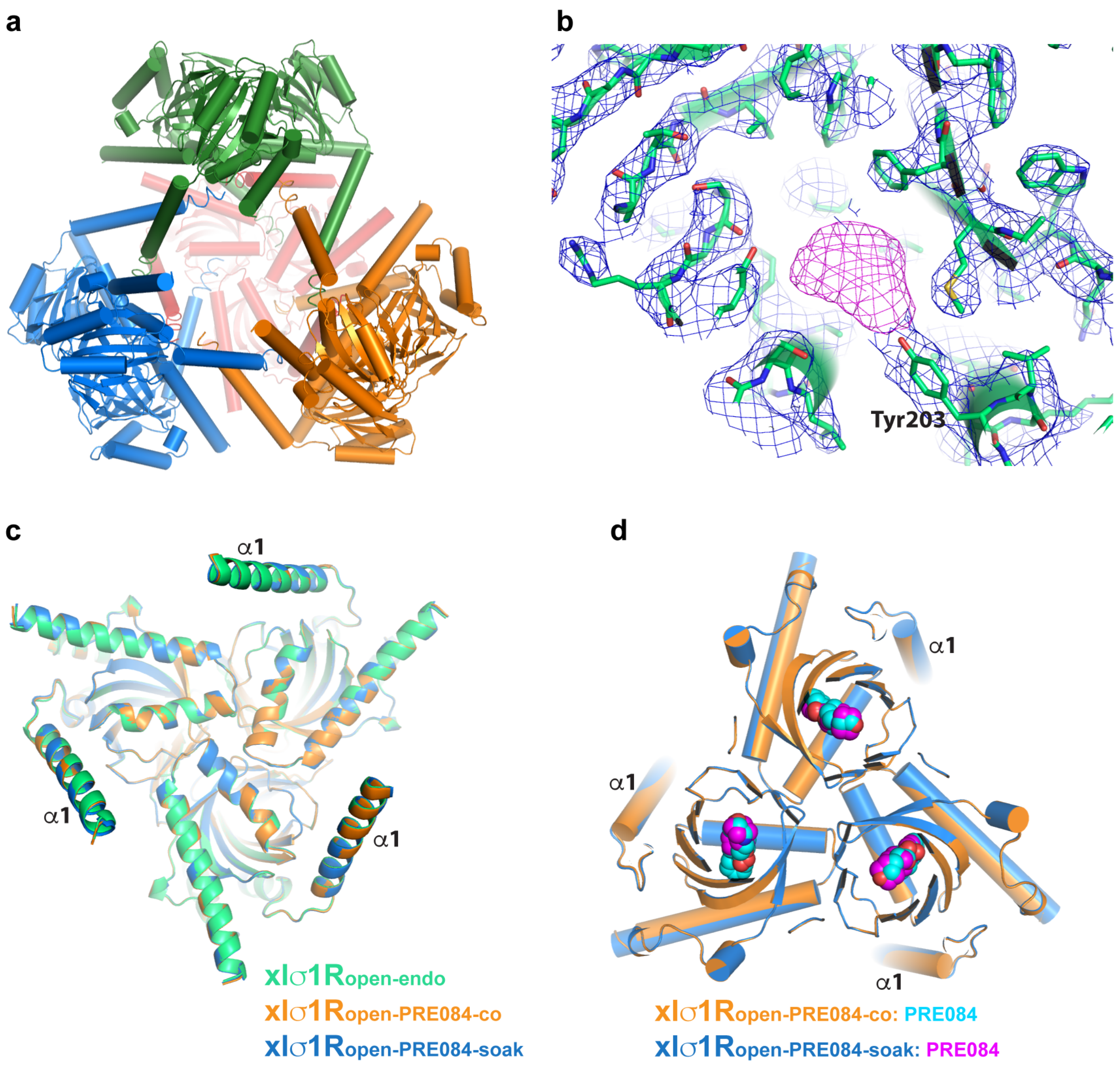

Supplementary Figure 3. The structures of xlσ1R in an open-like conformation. (a) Crystal packing of xlσ_1R$_{\text{open-endo}}$. The four homotrimers in one asymmetric unit are assembled in a tetrahedron shape and displayed in four colors. (b) The close-up view of the electron density map of one protomer of xlσ_1R$_{\text{open-endo}}$, which is displayed in both sticks and the cartoon mode, viewed parallel to the membrane. The blue mesh shows the simulated annealing 2F_o-F_c map contoured at 1.2 σ level, and the purple mesh shows the simulated annealing F_o-F_c map contoured at 3.0 σ level corresponding to an unidentifiable molecule. Tyr203 is labeled. (c) Superposition of the xlσ1R$_{\text{open-endo}}$ trimer (in green), the xlσ1R$_{\text{open-PRE084-co}}$ trimer (in orange), and

the xlσ1R$_{open\text{-}PRE084\text{-}soak}$ trimer (in blue), viewed perpendicular to the membrane from the membrane side. (d) Superposition of the xlσ1R$_{open\text{-}PRE084\text{-}co}$ trimer (in orange) and the xlσ1R$_{open\text{-}PRE084\text{-}soak}$ trimer (in blue), viewed perpendicular to the membrane from the cupin-fold side. The ligand, PRE084, is displayed in spheres in each protomer.

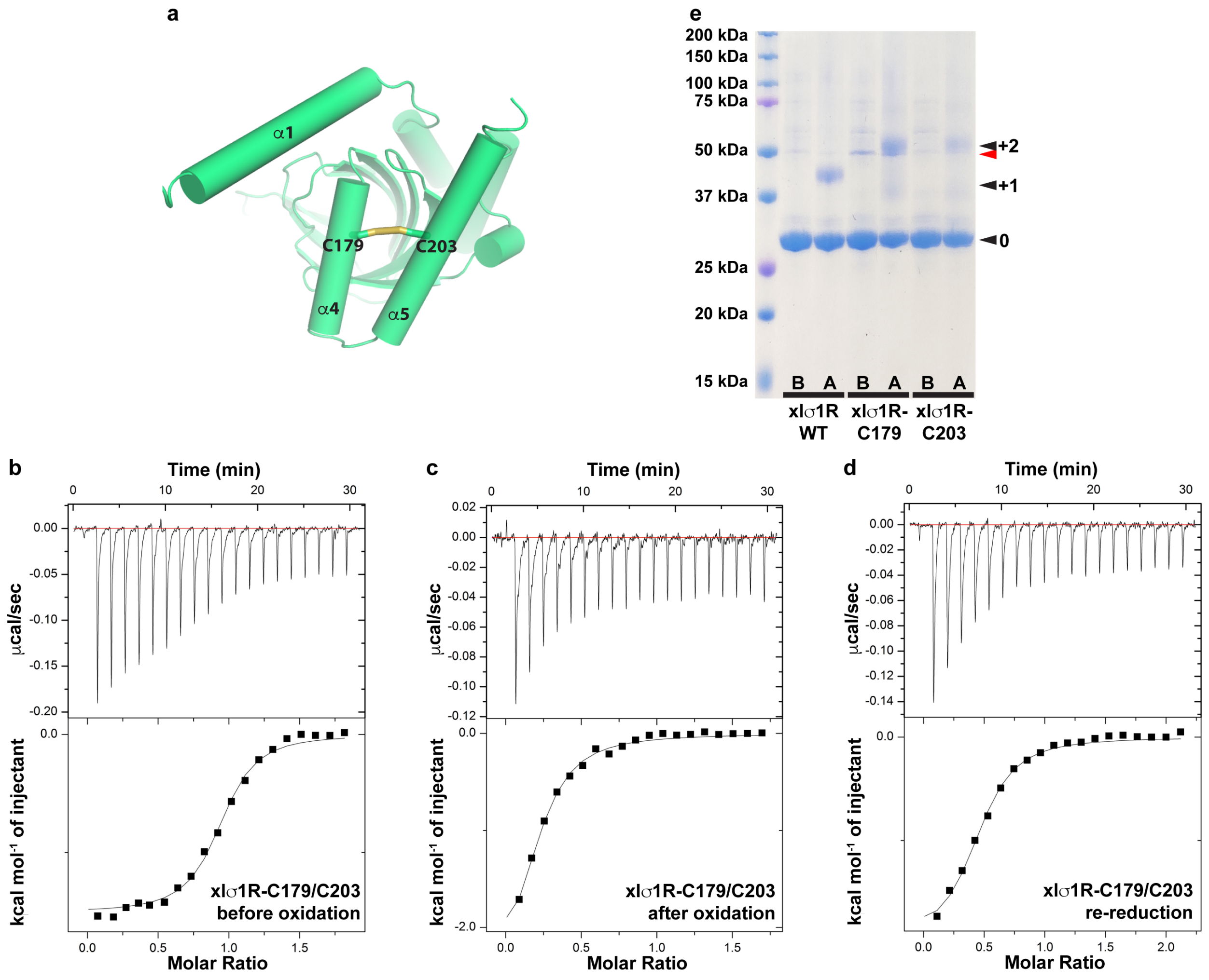

Supplementary Figure 4. Blocking the PATH2 entrance by targeting the residues 179 and 203 of xlσ1R. (a) The structure of one protomer of xlσ1R$_{\text{closed-endo}}$, viewed perpendicular to the membrane from the membrane side. A disulfide bond is modeled between the residues 179 and 203. (b)-(d) Representative ITC data for PRE084 binding to the xlσ1R-C179/C203 protein before oxidation (b), after oxidation (c), and after re-reduction of the oxidized sample (d). (e) Modification of mPEG-Mal-5K to the indicated xlσ1R proteins in the indicated conditions analyzed by SDS-PAGE and Coomassie blue staining. 'B', protein sample before modification; 'A', protein sample after modification. The wild-type (WT) xlσ1R served as the control. The black arrowheads labeled with 0, +1 and +2 indicate the band positions without, with one, and with two mPEG-Mal-5K modification(s). The red arrowhead indicates a minor impurity band near 50

kDa, which does not affect the gel analysis. Source data are provided at the end of the Supplementary Information file. The modification experiment was repeated three times independently with similar results.

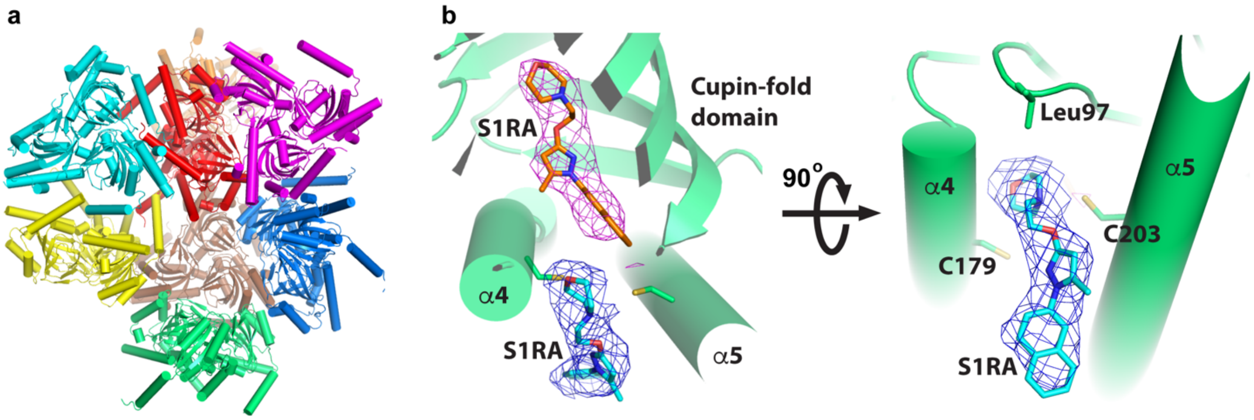

Supplementary Figure 5. The structure of xlσ1R$_{C179/C203\text{-}S1RA}$ captures a ligand potentially passing through the entrance. (a) Crystal packing of xlσ1R$_{C179/C203\text{-}S1RA}$. The eight homotrimers in one asymmetric unit are displayed in different colors. (b) The close-up view of the xlσ1R$_{C179/C203\text{-}S1RA}$ protomer containing two S1RA molecules, viewed parallel to the membrane (left panel) or perpendicular to the membrane from the membrane side (right panel). Leu97, Cys179, Cys203 and S1RA are displayed in sticks. The purple mesh shows the simulated annealing F_o-F_c omit map contoured at 3.0 σ level corresponding to the S1RA molecule in the ligand binding site, and the blue mesh shows the simulated annealing F_o-F_c omit map contoured at 3.0 σ level corresponding to the S1RA molecule passing through α4 and α5.

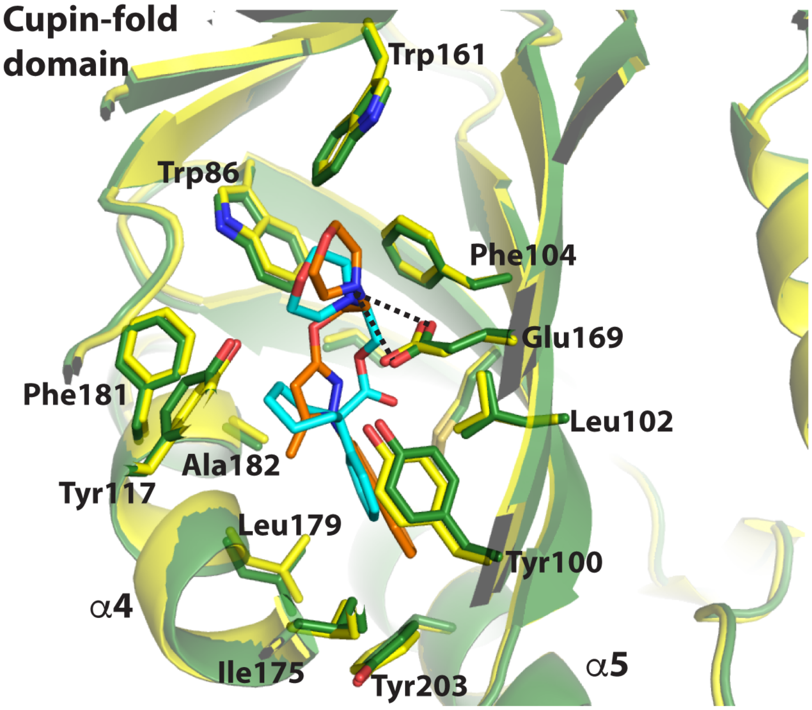

xlσ1R$_{\text{closed-PRE084}}$: PRE084
xlσ1R$_{\text{closed-S1RA}}$: S1RA

Supplementary Figure 6. The ligand binding site of xlσ1R. The close-up view of the superposition of one protomer of xlσ1R$_{\text{closed-PRE084}}$ (in green) and xlσ1R$_{\text{closed-S1RA}}$ (in yellow), viewed parallel to the membrane. The ligands, PRE084 (in cyan) and S1RA (in orange), are displayed in sticks. The residues of the ligand binding site are labeled and also rendered in sticks. The salt bridge between the cationic nitrogen of the ligand and the carboxy group of Glu169 is indicated by two black dashed lines.

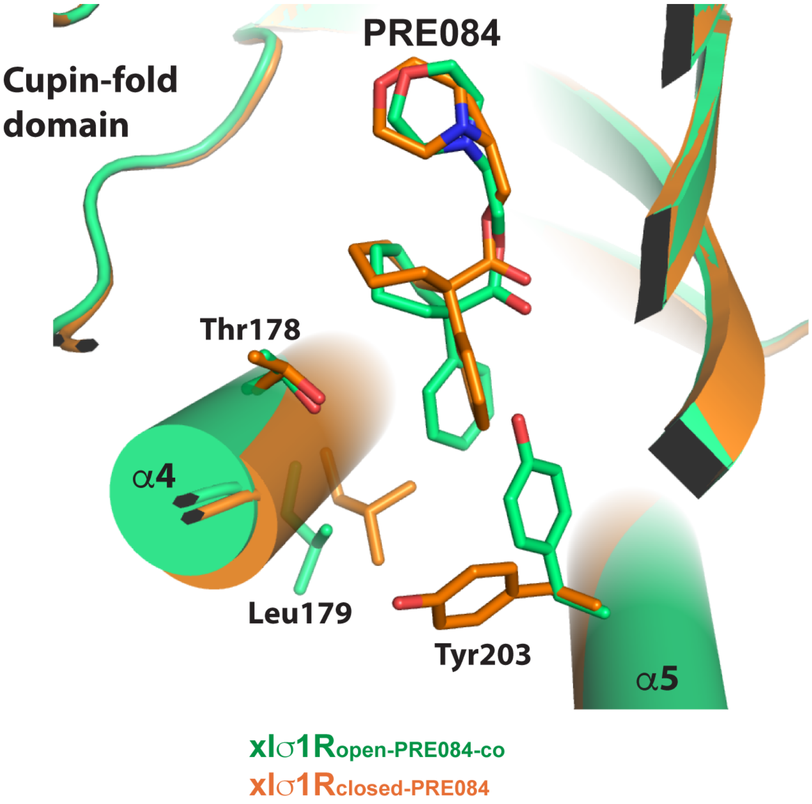

Supplementary Figure 7. Binding of a ligand, PRE084, in the closed and an open-like state of xlσ1R. The close-up view of the superposition of one xlσ1R$_{\text{open-PRE084-co}}$ protomer (in green) and one xlσ1R$_{\text{closed-PRE084}}$ protomer (in orange), viewed parallel to the membrane, showing the slightly different ligand poses, as well as the difference in α4/α5 between the two structures. Thr178, Leu179, Tyr203, and the ligand, PRE084, are rendered in sticks.

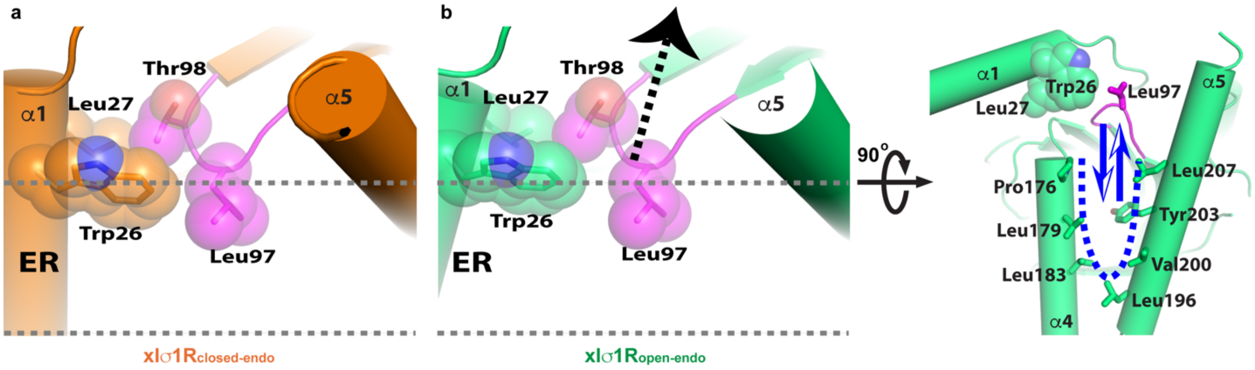

Supplementary Figure 8. A speculated role for the transmembrane helix, α1, during the ligand diffusion to the entrance. (a) The close-up view of one xlσ1R$_{closed\text{-}endo}$ protomer (in orange) showing the hydrophobic interactions between α1 and the Leu97-containing loop (in magenta) from the cupin-fold domain, viewed parallel to the membrane. The contacting residues are labeled and rendered in both sticks and spheres. The relative position of the ER membrane is indicated by two grey dashed lines. (b) Left, the close-up view of one xlσ1R$_{open\text{-}endo}$ protomer (in green) showing the same details as in panel (a). A black dashed arrow indicates a postulated swing-up of the Leu97-containing loop (in magenta) to move slightly away from the ER membrane. Right, the same xlσ1R$_{open\text{-}endo}$ protomer viewed perpendicular to the membrane from the membrane side. Trp26 and Leu27 of α1 are displayed in spheres. The putative entrance between α4 and α5 is indicated by a blue dashed curved line, and the entrance-surrounding residues are rendered in sticks. The Leu97-containing loop (in purple) is modeled to have swung away from the membrane, and two blue arrows indicate the diffusion of the ligand to and from the entrance center, while the ligand is dissolved within the membrane.

Supplementary References

1. Thompson, J.D., Higgins, D.G. & Gibson, T.J. CLUSTAL W: improving the sensitivity of progressive multiple sequence alignment through sequence weighting, position-specific gap penalties and weight matrix choice. *Nucleic Acids Res* **22**, 4673-80 (1994).
2. Combet, C., Blanchet, C., Geourjon, C. & Deleage, G. NPS@: network protein sequence analysis. *Trends Biochem Sci* **25**, 147-50 (2000).

Source Data for Supplementary Fig. 4e

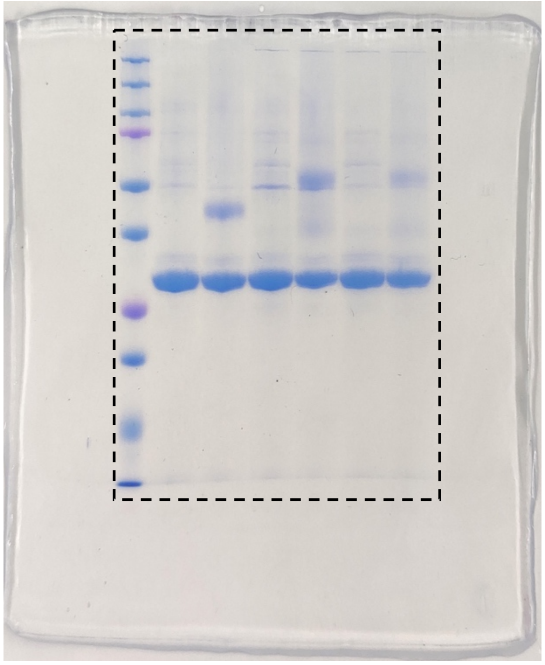

Supplement: Supplementary file 1 — Supplementary Information [file 41467_2022_28946_MOESM1_ESM.pdf]
