# Supplementary material for: An open-like conformation of the sigma-1 receptor reveals its ligand entry pathway
Source: Nat Commun. 2022 Mar 10;13:1267. doi: 10.1038/s41467-022-28946-w (PMC8913746; doi:10.1038/s41467-022-28946-w)
Supplement: Supplementary file 3 — Reporting Summary [file 41467_2022_28946_MOESM3_ESM.pdf]

nature portfolio

Corresponding author(s): Ziyi Sun, Xiaoming Zhou

Last updated by author(s): Jan 31, 2022

Reporting Summary

Nature Portfolio wishes to improve the reproducibility of the work that we publish. This form provides structure for consistency and transparency in reporting. For further information on Nature Portfolio policies, see our Editorial Policies and the Editorial Policy Checklist.

Statistics

For all statistical analyses, confirm that the following items are present in the figure legend, table legend, main text, or Methods section.

n/a	Confirmed	
☐	☒	The exact sample size (*n*) for each experimental group/condition, given as a discrete number and unit of measurement
☐	☒	A statement on whether measurements were taken from distinct samples or whether the same sample was measured repeatedly
☐	☒	The statistical test(s) used AND whether they are one- or two-sided<br>*Only common tests should be described solely by name; describe more complex techniques in the Methods section.*
☒	☐	A description of all covariates tested
☒	☐	A description of any assumptions or corrections, such as tests of normality and adjustment for multiple comparisons
☐	☒	A full description of the statistical parameters including central tendency (e.g. means) or other basic estimates (e.g. regression coefficient) AND variation (e.g. standard deviation) or associated estimates of uncertainty (e.g. confidence intervals)
☐	☒	For null hypothesis testing, the test statistic (e.g. *F*, *t*, *r*) with confidence intervals, effect sizes, degrees of freedom and *P* value noted<br>*Give P values as exact values whenever suitable.*
☒	☐	For Bayesian analysis, information on the choice of priors and Markov chain Monte Carlo settings
☒	☐	For hierarchical and complex designs, identification of the appropriate level for tests and full reporting of outcomes
☒	☐	Estimates of effect sizes (e.g. Cohen's *d*, Pearson's *r*), indicating how they were calculated

Our web collection on statistics for biologists contains articles on many of the points above.

Software and code

Policy information about availability of computer code

Data collection	For ITC measurement: MicroCal iTC200 1.26.3.
Data analysis	For X-ray diffraction data processing: autoPROC pipeline package 1.0.5, XDS BUILT 20210323, CCP4 v7.<br>For phasing, model building and display: Phaser 2.6.0, Phenix 1.13, COOT 0.8.9.1, PyMOL 2.1.1, HOLLOW 1.1.<br>For sequence alignment: ClustalW NPS@ web server.<br>For band intensity analysis: ImageJ 1.53e.<br>For ITC binding analysis: Origin 7, Microsoft Excel for Mac 2016.

For manuscripts utilizing custom algorithms or software that are central to the research but not yet described in published literature, software must be made available to editors and reviewers. We strongly encourage code deposition in a community repository (e.g. GitHub). See the Nature Portfolio guidelines for submitting code & software for further information.

Data

Policy information about availability of data

All manuscripts must include a data availability statement. This statement should provide the following information, where applicable:

- Accession codes, unique identifiers, or web links for publicly available datasets
- A description of any restrictions on data availability
- For clinical datasets or third party data, please ensure that the statement adheres to our policy

The atomic coordinates and structure factors of the xlsigma1R structures generated in this study have been deposited in the Protein Data Bank under the following accession codes: 7W2B (xlsigma1Rclosed-endo), 7W2C (xlsigma1Rclosed-PRE084), 7W2D (xlsigma1Rclosed-S1RA), 7W2E (xlsigma1Ropen-endo), 7W2F (xlsigma1Ropen-PRE084-co), 7W2G (xlsigma1Ropen-PRE084-soak), 7W2H (xlsigma1RC179/C203-S1RA). A previously reported hsigma1R structure complexed with

the antagonist PD144418 (hsigma1RPD144418) used in this study is available in the Protein Data Bank under accession code 5HK1. Source data are provided with this paper.

Field-specific reporting

Please select the one below that is the best fit for your research. If you are not sure, read the appropriate sections before making your selection.

☒ Life sciences ☐ Behavioural & social sciences ☐ Ecological, evolutionary & environmental sciences

For a reference copy of the document with all sections, see nature.com/documents/nr-reporting-summary-flat.pdf

Life sciences study design

All studies must disclose on these points even when the disclosure is negative.

Sample size	The sample size was estimated from similar ITC experiments from former publications. The exact n values used to calculate the statistics are provided in the figure legends.
Data exclusions	No data was excluded from analysis.
Replication	All experiments were performed independently at least 3 times unless stated differently in the figure legends. All attempts at replication were successful under the experimental conditions defined.
Randomization	Purified protein samples were randomly assigned to the control or treatment groups.
Blinding	Protein samples were purified by multiple researchers, and ITC measurements were performed and analyzed by different researchers to ensure reproducibility of the data.

Reporting for specific materials, systems and methods

We require information from authors about some types of materials, experimental systems and methods used in many studies. Here, indicate whether each material, system or method listed is relevant to your study. If you are not sure if a list item applies to your research, read the appropriate section before selecting a response.

Materials & experimental systems

n/a	Involved in the study	
☒	☐	Antibodies
☒	☐	Eukaryotic cell lines
☒	☐	Palaeontology and archaeology
☒	☐	Animals and other organisms
☒	☐	Human research participants
☒	☐	Clinical data
☒	☐	Dual use research of concern

Methods

n/a	Involved in the study	
☒	☐	ChIP-seq
☒	☐	Flow cytometry
☒	☐	MRI-based neuroimaging
